# Comparative effectiveness and safety of direct oral anticoagulants compared to warfarin in morbidly obese patients with acute venous thromboembolism: systematic review and a meta-analysis

**DOI:** 10.1007/s11239-020-02179-4

**Published:** 2020-06-18

**Authors:** Mohamed Nabil Elshafei, Mouhand F. H. Mohamed, Ahmed El-Bardissy, Mohamed Badie Ahmed, Ibtihal Abdallah, Hazem Elewa, Mohammed Danjuma

**Affiliations:** 1 Clinical Pharmacy Department, Hamad General Hospital, Doha, Qatar; 2 Internal Medicine Department, Hamad Medical Corporation, Doha, Qatar; 3 College of Medicine, QU Health, Qatar University, Doha, Qatar; 4 College of Pharmacy, QU Health, Qatar University, Doha, Qatar

**Keywords:** Pulmonary embolism, Warfarin, DVT, Morbid obesity, Overweight

## Abstract

Direct oral anticoagulant (DOAC) agents are becoming the anticoagulation strategy of choice. However, their use in the treatment of acute venous thromboembolism (VTE) in morbidly obese patients (bodyweight of > 120 kg or BMI > 40 kg/m^2^) guarded. This is due to the scarce data supporting their use in this population. As a result, the International Society on Thrombosis and Haemostasis recommended against their use in this cohort of patients. New data emerged supporting the use of DOACs in these patients. Hence, we aimed to systematically review the literature exploring the efficacy and safety of these agents compared to warfarin in VTE treatment in morbidly obese patients. A systematic review of PubMed and EMBASE since inception until 01/04/2020. Subsequently, a non-inferiority (NI of 1.75) meta-analysis utilizing the random-effects model. Five observational studies (6585 patients) were included in our meta-analysis. DOAC analogs were non-inferior compared to warfarin in reducing the primary efficacy outcome of VTE recurrence (OR 1.07, 95% CI 0.93–1.23) and the primary safety outcome (major bleeding events) (OR 0.80, 95% CI 0.54–1.17). Our meta-analysis comprising real-world observational data concludes that the use of DOAC analogs in morbidly obese patients (bodyweight of > 120 kg or BMI > 40 kg/m^2^) is non-inferior with regards to efficacy and safety compared to warfarin. This finding helps to resolve the uncertainty associated with the use of DOACs in this cohort. Additionally, it invites for a confirmatory non-inferiority randomized controlled trial testing DOAC vs. Warfarin in this group of patients.

**Electronic supplementary material:**

The online version of this article (10.1007/s11239-020-02179-4) contains supplementary material, which is available to authorized users.

## Highlights

- The use of DOACs in the treatment of acute venous thromboembolism (VTE) in morbidly obese patients is controversial.
- There are concerns of decreased drug exposure and under-dosing in the obese patients’ population.
- This meta-analysis, aimed to evaluate the effectiveness (rates of VTE events) and safety (major bleeding) of DOAC analogs compared to warfarin in patients with extremely high body weight with acute VTE.
- The project concludes that the use of DOACs in morbidly obese patients (bodyweight of > 120 kg or BMI > 40 kg/m^2^) is effective and safe. It supports the current practice of using DOAC analogs as an alternative to warfarin in this cohort of patients.

## Introduction

Venous thromboembolism (VTE) is a prevalent clinical entity affecting approximately 1 to 2 per 1000 patients [1]. These events primarily involve the deep veins of the lower extremities causing deep vein thrombosis (DVT), or embolize to the pulmonary arteries causing a pulmonary embolism (PE). Untreated, PE is associated with a mortality rate that reaches up to 30% compared with 2–11% in those treated with anticoagulation (AC) [2–4]. Therefore, prompt identification and treatment initiation with AC therapy is imperative.

Obesity is a worldwide epidemic that drives increasing morbidity and mortality from thrombotic disorders, such as myocardial infarction, stroke, and VTE. It is considered a significant risk factor for VTE by enhancing blood stasis. Studies have shown a significantly increased risk for DVT and PE in this group of patients [5–7].

In patients with VTE, AC is mandatory to prevent thrombus propagation and recurrence. Low-molecular-weight heparin (LMWH) followed by oral anticoagulation with vitamin K antagonists (VKA) has been considered the mainstay of therapy until a few years ago [8]. However, as evidenced in variable settings where AC is utilized, warfarin therapy is fraught with a lot of clinical, therapeutic, and logistical issues. These range from potential drug–drug and drug-food interactions, inter and intra-individual variability in both responses to treatment and risk of side effects. Others include the logistics of reliable and robust International normalizing ratio (INR) monitoring [9–12]. Consequent upon these, direct oral anticoagulants (DOACs) have been developed, including factor IIa (thrombin) and factor Xa inhibitors, and introduced to the market. They are approved by the food & drug administration (FDA) for the management of acute VTE. A steady stream of randomized controlled clinical trials has demonstrated the non-inferiority of these agents when compared to VKA in terms of both efficacy and safety, to reduce of risk of recurrence in patients with both DVT and PE [13–17]. This has resulted in their incorporation into therapeutic national/society guidelines [18]. Since the introduction of DOACs to the market, AC management had encountered significant changes. Current guidelines such as CHEST guidelines suggest DOACs over warfarin in both non-valvular atrial fibrillation (AF) and non-cancer VTE patients [18, 19].

Furthermore, DOACs have a wider therapeutic window at fixed dosing regimens, in addition to minimal and manageable food and drug interactions with no requirement for routine monitoring. However, the low representation of obese patients, particularly those with morbid obesity, in the major trials has raised questions about the efficacy, adequacy of fixed dosing, and safety of direct oral anticoagulants in these cohorts of patients. None of the RCTs reported the results of patients with morbid obesity (BMI ≥ 40 kg/m^2^) [13–17].

There is a scarcity of evidence investigating the efficacy of DOACs in obese patients. In a study of apixaban, bodyweight of more than 120 kg and body-mass index [BMI] ≥ 30 kg/m^2^ were associated with a lower mean peak concentration and higher volume of distribution compared with average weight [20]. On the contrary, peak concentration, distribution, and half-life of rivaroxaban were similar between patients who weighed more than 120 kg and those who weighed 70–80 kg [21]. Therefore, because of the concerns of decreased drug exposure and under-dosing in the obese patients’ population, the Scientific and Standardization Committee of the International Society on Thrombosis and Hemostasis in its 2016 clinical practice guidelines recommended against the use of DOACs in patients with a BMI of more than 40 kg/m^2^ or weighing more than 120 kg [22]. It also suggested Obtaining drug-specific peak and trough levels to assess therapeutic appropriateness in this population if DOACs are prescribed. Therefore, there is an unresolved uncertainty regarding the utility of DOAC analogs as an acute VTE treatment strategy in morbidly obese patients (BMI > 40 kg/m^2^ or weight > 120 kg). It will be valuable to demonstrate that DOACs are at least non-inferior to VKA in terms of efficacy and safety in this patient population.

Our meta-analysis, therefore, aimed to evaluate the effectiveness (rates of VTE events) and safety (major bleeding) of DOAC analogs compared to warfarin in patients with extremely high body weight with acute VTE.

## Methods

This review followed PRISMA guidelines [23].

### Study eligibility criteria

We attempted to include both real-world observational data and randomized controlled trials that compared DOAC analogs to warfarin in morbidly obese patients (BMI > 40 or weight > 120 kg). At a minimum, studies assessed VTE recurrence or major bleeding events to be included in our review. We excluded studies reporting on pediatrics (< 18 years old), as well as studies failing to meet the inclusion criteria.

### Search strategy

We performed an exhaustive literature search of PubMed, Medline, and EMBASE since their inception till 01/04/2020. No language, date, or article type limitations were adopted in our search strategy. Example of a database search strategy is: ((((((((((((direct oral anticoagulants) OR (DOAC)) OR (DOACs)) OR (NOAC)) OR (NOACs)) OR (Novel oral anticoagulants)) OR (Rivaroxaban)) OR (Apixaban)) OR (Edoxaban)) OR (Dabigatran)) OR (betrixaban)) AND ((((((((((deep venous thromboses[MeSH Terms]) OR (pulmonary embolism[MeSH Terms])) OR (venous thromboembolism[MeSH Terms])) OR (venous thrombosis)) OR (Venous thromboembolism)) OR (VTE)) OR (DVT)) OR (PE)) OR (Deep venous thrombosis)) OR (Pulmonary embolism))) AND ((((((((obese) OR (obesity)) OR (overweight)) OR (Morbid obese)) OR (high BMI)) OR (central obesity[MeSH Terms])) OR (morbid obesity[MeSH Terms])) OR (morbid obesities[MeSH Terms])). Additionally, we attempted a manual reference search of retrieved studies.

### Screening and data extraction

Title and abstract screening were attempted initially. Eligible articles were retrieved for full-text review and assessment for inclusion in our review. Two reviewers (MNE and MFHM) conducted the search and screening. In the case of disagreement not settled by discussion, a third reviewer (AEB) adjudicated the disagreement following the protocol. We used a preplanned template to extract the data. Examples of the data extracted are; general articles information such as the author, publication year, study design, intervention, control, outcome, BMI, weight. Etc.

### Outcome

The primary outcome in our review is the rate of VTE recurrence. Major bleeding events served as our secondary outcome (as defined by the primary study authors). We would look at these outcomes at 6 months of follow-up whenever specified in the study, otherwise the longer duration of observation if no specification provided.

### Study quality and risk of bias assessment

We planned to utilize the Cochrane Collaboration’s tool for assessing the risk of bias and quality of randomized controlled trials [24]. Additionally, we used the New castle Ottawa tool to assess the risk of bias assessment of observational studies [25]. We generated funnel plots to screen for publication bias.

### Statistical analysis

The odds ratios (OR) were computed as a measure of effect size. The Forest plot was generated to summarize the results. Additionally, we conducted a sensitivity analysis to screen for consistency and small-study effects. The I^2^ statistic was used to report heterogeneity. An I^2^ > 50% is suggestive of marked heterogeneity in our review. The random-effects model was used as our meta-analytical technique. Understanding that there might be a paucity of studies, we opted for a non-inferiority (NI) margin that corresponds to an OR of 1.75. This NI margin was generated based on a systematic review by Prins et al., and is less than what was used in the EINSTEIN-PE study (NI = 2), and corresponds to the preservation of at least 75% of the effect of warfarin over placebo [26, 27]. MetaXl software was used for statistical analysis (version 5.3 © EpiGear International Pty Ltd ABN 51 134 897 411 Sunrise Beach, Queensland, Australia, 2011–2016).

## Results

**Fig. 1:**
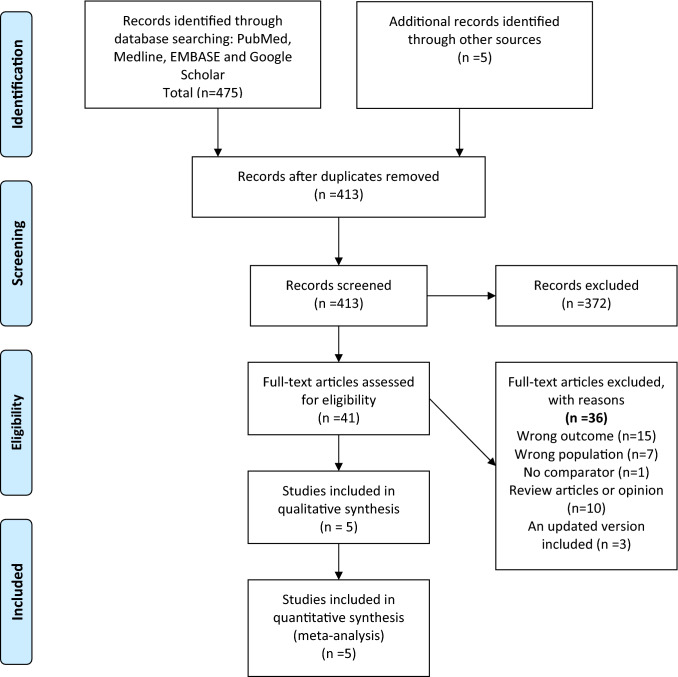
PRISMA flow diagram

Our exhaustive search strategy retrieved 475 titles. After screening, five studies were included in our final analysis (Fig. 1 shows the PRISMA flow diagram) [28–32]. The total number of patients evaluated in these studies is 6575 patients. A real-world registry-based retrospective cohort study contributed to the majority of the patients (5780 patients) [32]. All the studies were observational, with the absence of randomized controlled studies meeting our eligibility criteria. Four studies evaluated each of our primary efficacy (VTE recurrence) and safety (major bleeding) outcomes. One study was excluded from the primary efficacy analysis as it reported VTE recurrence in rates [30]. Another study was excluded from the safety outcome as it reported only a composite of major bleeding events and clinically relevant-non-major bleeding events [29]. (Table 1 summary of studies included in the meta-analysis).

**Table 1:** Summary of included studies

Author Study design	Number of patients (n)	Ethnicity	Mean age (SD)	Male n (%)	BMI (kg/m^2^) or weight (kg) median [IQR]	BMI ≥ 50 N(%)	Included and upper limit of BMI	Follow-up period	Included efficacy outcome	Included safety outcome
Kushnir 2019 Single center Retrospective	T: (n = 366) Rivaroxaban (n = 152) Apixaban (n = 47) Warfarin: (n = 167)	T: White (16%) Black (47%) Others/unknown (37%)	55·1 (15·0) Rivaroxaban 52·6 (14·5) Apixaban 53.3 (13.9) 58·1 (15·1)	113 (31%) Rivaroxaban 52(34%) Apixaban 12 (25%) 49 (29%)	44·7(kg/m^2^), [41·3–50·1] Rivaroxaban 43·7(kg/m^2^), [41·1–48·8], Apixaban 43.3 (kg/m^2^), [41.2–49.4] 45.3 (kg/m^2^), [41.4–52.5]	92 (25%) Rivaroxaban 30 (20%), Apixaban 10 (21%) 52 (31%)	Included:BMI ≥ 40, Upper limit BMI: 88	196 [89.4–457.3] days	Recurrent VTE, stroke	Major bleeding based on ISTH definition Clinical Non major bleeding
Spyropoulos 2019 Retrospective cohort study	Rivaroxaban (n = 2890) Warfarin (n = 2890)	NR	53.3 (12.9) 53.1 (13.1)	1141 (39.5%) 1150 (39.8%)	Diagnostic codes of morbid obesity (ICD 9 & 10 codes)		Using claim-data base ICD codes Upper limit BMI: NR	≥ 3 months	Recurrent VTE	Major bleeding
Perales 2019^a^ Retrospective	T: (n = 176) Rivaroxaban (n = 84) Warfarin (n = :92)	NR	56 (14.5) 56 (14) 55 (15)	95 (54%) 44 (52%) 51 (55%)	45 [41–50] 45 [41–51] 44 [41–50]		Included: BMI > 40 or weight > 120 kg Upper limit BMI: NR	12 months	VTE recurrence, stroke incidence, and mortality	Length of stay and bleeding complications
Quan 2020^c^ Retrospective	T: (n = 187) DOAC: (n = 109) Traditional therapy^d^: (n = 78)	NR	53 (42–61) 53 (43–61) 52 (42–61)	122 (65.2%) 72 (66.0%) 50 (64.1%)	140 [130–157] 138 [129–154] 142 [130–161]		Weight > 120 kg Upper limit BMI: NR	12 months	Recurrent VTE,AC regimens (agent/dosing)	Bleeding events
Almeida 2019 Retrospective	T: (n = 133) DOACs: (n = 71) Warfarin: (n = 62)	NR	NR	NR	Weight ≥ 120 kg^b^		Weight ≥ 120 kg^b^, Upper limit BMI: NR	12 months	Recurrent VTE	Bleeding events

Data are presented as mean ± SD, median [interquartile range]

*T* total number of patients, *BMI* body mass index, *IQR* interquartile range, *NR* not reported, *DOAC* direct oral anticoagulant, *AC* Anticoagulation

^a^Was excluded from safety analysis as it reported the composite bleeding events only

^b^The study analyzed (BMI ≥ 30 kg/m^2^ versus < 30 kg/m^2^) and body weight (≥ 120 kg vs. < 120 kg) but we included only body weight ≥ 120 kg

^c^Was excluded from efficacy analysis as it reported VTE recurrence in rates

^d^Traditional therapy, LMWH ± Warfarin

### Recurrent venous thromboembolism

**Fig. 2:**
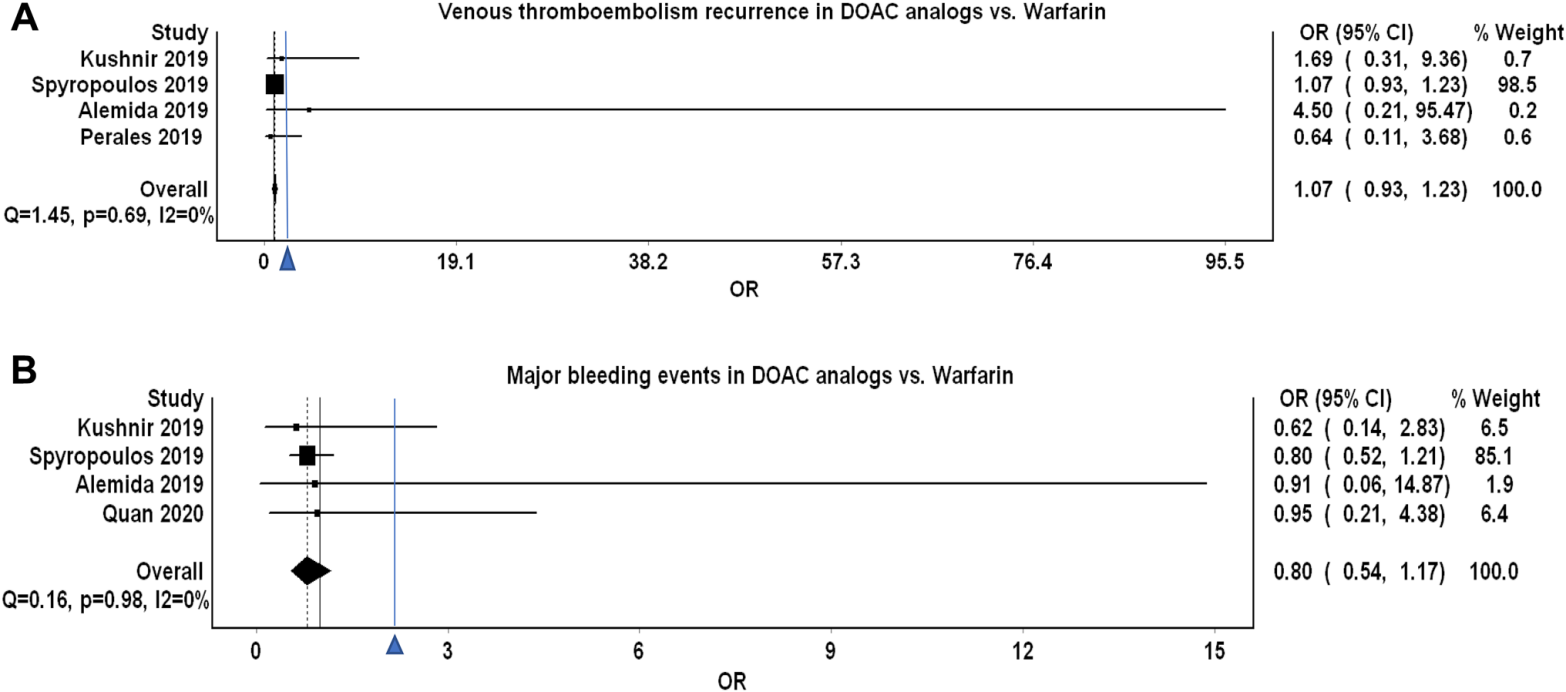
**a** Depicting a forest plot of VTE recurrence rates in DOAC analogs compared to warfarin in morbidly obese patients. **b** Depicting a forest plot of major bleeding events in DOAC analogs compared to warfarin in morbidly obese patients

**Fig. 3:**
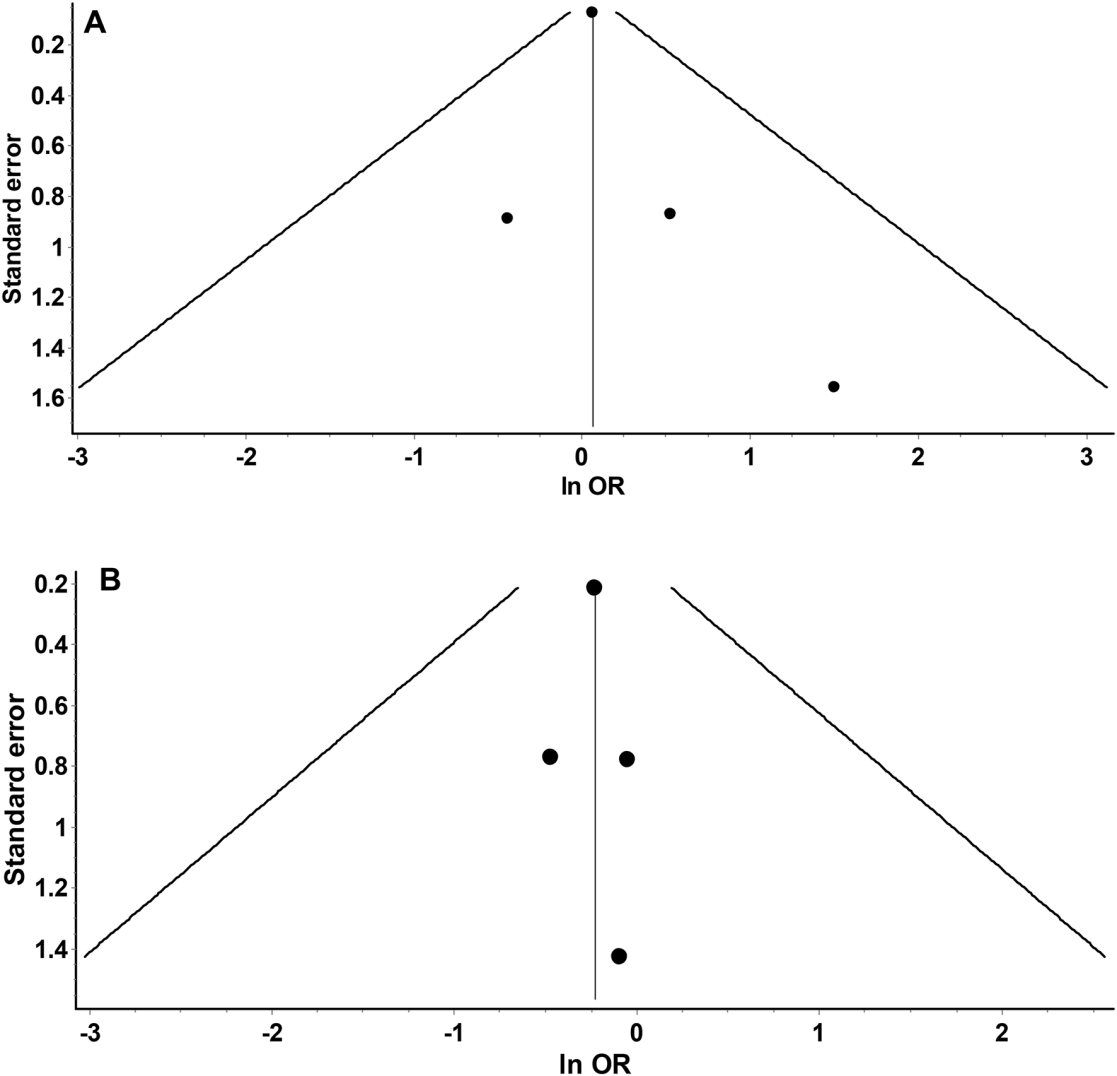
**a** Funnel plot to assess the publication bias for studies assessing VTE recurrence in DOAC analogs vs. warfarin displaying no marked asymmetry. **b** Funnel plot to assess the publication bias for studies assessing major bleeding events in DOAC analogs vs. warfarin showing no marked asymmetry

Four studies evaluated VTE recurrent events in morbidly obese patients [28, 29, 31, 32]. These studies showed that DOAC analogs were non-inferior with regards to the primary efficacy outcome of VTE recurrent events (OR 1.07, 95% CI 0.93–1.23), Q 1.45, I2 0%. The low I^2^ suggested the homogeneity of the results (Fig. 2). The funnel plot revealed no marked asymmetry (Fig. 3). Sensitivity analysis showed overall consistency in the final point estimate upon ordered exclusion of the constituent studies; nonetheless, excluding the most extensive study led to a widening of the confidence interval with upper bound crossing the non-inferiority margin (Supplementary material Table 1) [32].

### Major bleeding

Four observational studies evaluated and reported the risk of major bleeding events [28, 30–32]. DOAC analogs had a consistent non-significant trend towards an overall reduced risk of major bleeding events by 20% (OR 0.80, 95% CI 0.54–1.17, Q = 0.16, I^2^ = 0%) (Fig. 2). The funnel plot showed no marked asymmetry, however, limited by a small number of studies (Fig. 3). Sensitivity analysis did not affect the final point estimate. The exclusion of Spyropoulos et al. resulted in only the widening of the CI (Pooled OR 0.78, 95% CI 0.28–2.14, Q = 0.16, I^2^ = 0%) (Supplementary material Table 1).

### Risk of bias assessment

Most of the included studies were of moderate to high quality (NOS > 7) (Supplementary material Table 2 shows a quality assessment of the studies included in the review). The funnel plot showed moderate asymmetry (limited by the small number of trials) (Table 3). Hence, publication bias cannot be ruled out.

## Discussion

Obesity is an independent risk factor for the acquisition of VTE. Extremely high BMI has been shown to be correlated with an increased incidence of VTE; this correlation is more apparent with a BMI of 30 kg/m^2^ or more [33, 34]. The increased risk of VTE in this cohort is likely due to the increased abdominal pressure and the mechanical effect it exerts on the veins [35, 36]. Furthermore, the associated molecular hypercoagulable status; this status is postulated to be due to the associated elated levels of tumor necrosis factor-alpha (TNF-α), transforming growth factor-beta (TGF-β) [35, 36]. Moreover, the increased levels of Von Willebrand factor, and clotting factors, such as factor VII, factor VIIIc, and fibrinogen [37–39].

The clinical implications (efficacy and safety) of such observations remain uncertain. Subgroup analysis of randomized controlled trials of DOACs in VTE treatment have shown that their efficacy in obese patients (> 100 kg) had no difference compared to average weight [17, 40–42]. However, morbidly obese (bodyweight of > 120 kg or BMI > 40 kg/m^2^) patients were significantly under-represented in these trials. These inconsistencies were the driving factor behind the broad statement of the ISTH recommending against the use of DOACs in extremely high body weight (body weight of > 120 kg or BMI > 40 kg/m^2^) [22].

Our meta-analysis, aimed at settling this uncertainty, demonstrated that DOAC analogs are non-inferior to warfarin in terms of effectiveness (VTE events) in morbidly obese patients. Additionally, it showed a propensity towards lower major bleeding events. To the best of our knowledge, our meta-analysis is the first to address the uncertainty regarding the efficacy and safety of DOACs in patients with VTE and extremely high body weight [40–42]. The low I_2_ and the results of the sensitivity analysis indicated the homogeneity of our data. All the studies included in our review are relatively recent (2019 and 2020). Hence, these were not available to previous reviewers attempting to resolve this uncertainty.

The registry-based study by Spyropoulos et al. 2019 makes up to 88% of patients included in our review. They retrospectively studied rivaroxaban compared to warfarin in an adjusted comparison of 5780 patients. From this real-world analysis, they concluded a similar efficacy (rates of VTE events) and safety (major bleeding events). One major limitation of this study was the use of a claims-coded database. Besides, it did not report the International Normalized Ration (INR), and the time in therapeutic (TTR) for patients on warfarin, thus, bias may have been introduced [32]. Kushiner et al. 2019 investigated the use of rivaroxaban and Apixaban vs. warfarin in morbidly obese patients (BMI of ≥ 40 kg/m^2^) with atrial fibrillation and DVT. This study included 366 patients and also concluded that the incidence of recurrent VTE and major bleeding did not differ across the three cohorts [28]. This study was limited by missing data for patients’ history of thrombotic risk factors and by the presence of malignancy and bariatric surgery, which might independently contribute to a higher risk of thromboembolism. Additionally, a high proportion of the population was of African American and Hispanic origin, which questions the generalizability of the findings to other racial groups of morbidly obese patients [28].

In early 2020, Coons et al. retrospectively evaluated VTE recurrence and bleeding outcomes in 1840 cases of acute VTE, which were treated with either DOACs or warfarin. Included patients had bodyweight that ranged between 100 and 300 kg. This study did not detect any significant difference in the rate of VTE recurrence between DOACs and warfarin (6.5% vs. 6.4%; p = 0.93). Bleeding occurred in 1.7% and 1.2% of patients on DOACs and warfarin, respectively (p = 0.31). However, 50–55% of the patients in this study had a BMI of less than 40 kg/m^2^. Although their results support our conclusion, they did not report the outcomes for morbidly obese patients exclusively; hence, their study was excluded from our review [43].

Our meta-analysis is the first meta-analysis that demonstrated the non-inferior effectiveness and safety of DOAC analogs in morbidly obese patients and resolved this uncertainty. It has a good number of patients, out of which the biggest is a registry-based study examining the effect of these agents in real-world settings. Our review is not without limitations. It comprised of observational studies only; it is known that these studies have an inherently higher risk of bias. Secondly, we did not adjust for potential confounders (age, gender, and history of-or active malignancy). Additionally, the major DOAC used in the included studies was rivaroxaban, followed by Apixaban. This limits the generalizability of our findings to other DOAC analogs. Lastly, DOACs dosing information including drug interactions were not reported except for Quan et al. [30].

Acknowledging these limitations, a multicenter randomized controlled trial testing DOAC analogs vs. warfarin in morbidly obese patients is needed to settle this uncertainty once and for all. We think that the use of DOAC analogs as an intervention in this study will be ethically justifiable by the results of our review and the primary studies included in it.

## Conclusion

Our meta-analysis concludes that the use of DOACs in morbidly obese patients (bodyweight of > 120 kg or BMI > 40 kg/m^2^) is effective and safe. It supports the current practice of using DOAC analogs as an alternative to warfarin in this cohort of patients. However, to finally settle this dispute and to support our findings, a randomized controlled trial to confirm the non-inferiority of DOAC analogs vs. warfarin in morbidly obese patients is warranted.

## Electronic supplementary material

Below is the link to the electronic supplementary material.

Supplementary material 1 (DOCX 15 kb)

Supplementary material 2 (DOCX 23 kb)
