# Supplementary material for: Comparative effectiveness and safety of direct oral anticoagulants compared to warfarin in morbidly obese patients with acute venous thromboembolism: systematic review and a meta-analysis
Source: J Thromb Thrombolysis. 2020 Jun 18;51(2):388–96. doi: 10.1007/s11239-020-02179-4 (PMC7886743; doi:10.1007/s11239-020-02179-4)
Supplement: Supplementary file 1 — Supplementary material 1 (DOCX 15 kb) [file 11239_2020_2179_MOESM1_ESM.docx]

**TableS 1-A:** Table showing the sensitivity analysis of studies assessing VTE recurrence in DOACs vs. warfarin

| Excluded study | Pooled OR | LCI 95% | HCI 95% | Cochran Q | p | I 2 |
| --- | --- | --- | --- | --- | --- | --- |
| Kushnir 2019 | 1.0678077 | 0.9292963 | 1.2269643 | 1.1734529 | 0.5561449 | 0 |
| Spyropoulos +2019 | 1.2862478 | 0.4142059 | 3.9942293 | 1.3483721 | 0.509571 | 0 |
| Alemida 2019 | 1.0678794 | 0.9296504 | 1.2266616 | 0.601587 | 0.7402306 | 0 |
| Perales 2019 | 1.0745052 | 0.9351403 | 1.2346397 | 1.1212412 | 0.5708547 | 0 |

+We note that the Spyropoulos is driving the effect of this review. With its exclusion the confidence interval widens, however, with no significant change to the point estimate.

**TableS 1-B:** Table showing the sensitivity analysis of studies assessing major bleeding events in DOACs vs. warfarin

| Excluded study | Pooled OR | LCI 95% | HCI 95% | Cochran Q | p | I 2 |
| --- | --- | --- | --- | --- | --- | --- |
| Kushnir 2019 | 0.809139439 | 0.542410505 | 1.207031624 | 0.055563985 | 0.972600378 | 0 |
| Spyropoulos 2019 | 0.786034869 | 0.288500821 | 2.141591188 | 0.161392819 | 0.922473703 | 0 |
| Alemida 2019 | 0.793378416 | 0.536949677 | 1.17226872 | 0.152885646 | 0.926405882 | 0 |
| Quan 2020 | 0.785709598 | 0.526845475 | 1.171765919 | 0.104899925 | 0.9489018 | 0 |

+We note that the Spyropoulos is driving the effect of this review. With its exclusion the confidence interval widens, however, with no significant change to the point estimate being consistent to be in favor of DOACs.
