# Supplementary material for: Comparative effectiveness and safety of direct oral anticoagulants compared to warfarin in morbidly obese patients with acute venous thromboembolism: systematic review and a meta-analysis
Source: J Thromb Thrombolysis. 2020 Jun 18;51(2):388–96. doi: 10.1007/s11239-020-02179-4 (PMC7886743; doi:10.1007/s11239-020-02179-4)
Supplement: Supplementary file 2 — Supplementary material 2 (DOCX 23 kb) [file 11239_2020_2179_MOESM2_ESM.docx]

| **Study/ year** | **Selection** | **Comparability** | **Outcome** | **Total** |
| --- | --- | --- | --- | --- |
| Kushnir 2019 | * * * * | * * | * * | 8 |
| Spyropoulos 2019 | * * * * | * * | * * | 8 |
| Perales 2019 | * * * | * * | * * * | 8 |
| Quan 2020 | * * * | * * | * * * | 8 |
| Almeida 2019 | * * * | * * | * * * | 8 |

**Table S2:** NOS for the assessment of the included studies quality
